# The MicroRNA-21 in Autoimmune Diseases

**DOI:** 10.3390/ijms17060864

**Published:** 2016-06-03

**Authors:** Shaowen Wang, Xiaochun Wan, Qingguo Ruan

**Affiliations:** Shenzhen Laboratory of Fully Human Antibody Engineering, Institute of Biomedicine and Biotechnology, Shenzhen Institutes of Advanced Technology, Chinese Academy of Sciences, Shenzhen 518055, China; wangsw915918@163.com (S.W.); xc.wan@siat.ac.cn (X.W.)

**Keywords:** miR-21, autoimmune disease, Th17, apoptosis

## Abstract

MicroRNA-21 (miR-21) is an oncomiR and significantly upregulated in a wide range of cancers. It is strongly involved in apoptosis and oncogenesis, since most of its reported targets are tumor suppressors. Recently, miR-21 was found to be correlated with the pathogenesis of autoimmune diseases and may play an essential role in regulating autoimmune responses. In particular, miR-21 promotes Th17 cell differentiation, which mediates the development of multiple autoimmune diseases. In this article, we review the current research on the mechanisms that regulate miR-21 expression, the potential of miR-21 as a diagnostic biomarker for autoimmune disease and the mechanisms by which miR-21 promotes the development of autoimmune disease. We also discussed the therapeutic potential of targeting miR-21 in treating patients with autoimmune disease.

## 1. Introduction

MicroRNAs (miRNA) are small non-coding RNA molecules about 22 nucleotides in length. They are found in a wide range of eukaryotic species and function in RNA silencing and post-transcriptional regulation of gene expression. The gene encoding miRNA are usually transcribed by RNA polymerase II and processed by RNAse III enzyme Drosha to form an approximately 70 nucleotide precursor miRNA [1]. This precursor is then shuttled into the cytoplasm and forms a mature miRNA of approximately 22 nucleotides following processing by a second RNAse III enzyme called DICER. The mature miRNA is then incorporated into the RNA-induced silencing complex (RISC), which is able to recognize the “seed sequence” of the miRNA in target mRNA and negatively regulate its expression. Each miRNA is able to regulate the expression of several (maybe even hundreds of) target genes and are involved in important processes, such as embryonic development, immune response, inflammation, and oncogenesis, as well as cellular growth and proliferation [2]. The expression of miR-21 is significantly increased in a number of solid tumors such as breast, colon, gastric, and pancreatic cancers. Recent studies have revealed that miR-21 also plays a critical role in the regulation of immune system function [3]. miR-21 maintains low expression levels in non-activated T cells and antigen presenting cells (APC). However, its expression is significantly upregulated in these cells following activation [4,5,6]. Emerging studies indicate that miR-21 promotes inflammation and plays important roles during the pathogenesis of autoimmune disease including type 1 diabetes (T1D), psoriasis, multiple sclerosis (MS), rheumatoid arthritis (RA), and systemic lupus erythematosus (SLE).

## 2. The Regulation of miR-21

miR-21 is over-expressed in a variety of tumor cells. In addition, the expression of miR-21 can also be detected in mature or activated bone-marrow-derived mast cells, neutrophils, dendritic cells (DC), T cells, and B cell precursors. The expression of miR-21 is tightly regulated by a variety of extracellular and intracellular signaling molecules (Table 1). All-trans retinoic acid, phorbol 12-myristate 13-acetate (PMA), granulocyte-macrophage colony-stimulating factor (GM-CSF), IL-4, and lipopolysaccharide (LPS) can induce the expression of miR-21 in monocytes [6]. Therefore, miR-21 can serve as an important marker for immune cell activation. Further studies found that LPS, PMA, and IL-6 induce the expression of miR-21 through the activation of transcription factors NF-κB, AP-1 and STAT-3. Our group has also shown that p65, one of the five members of the NF-κB family, can bind to two distinct sites within the promoter region of miR-21 and positively regulate miR-21 expression [7]. In addition, expression of miR-21 was also regulated by a few negative regulators. Sawant *et al.* found that transcription repressor Bcl-6 competes with STAT3 for binding to the miR-21 promoter and thereby inhibits the miR-21 expression [8]. A recent study illustrated that lncRNA GAS5 (growth arrest specific 5), which belongs to the long non-coding RNA family, negatively regulates miR-21 expression [9]. PAP-associated domain-containing protein 5 (PAPD5), a non-canonical poly (A) polymerase, can mediate the adenylation of the 3′ end of miR-21 and cause the degradation of miR-21 [10]. The expression of miR-21 is regulated by multiple signaling molecules. How these molecules coordinate with each other to promote or inhibit the expression of miR-21 and how miR-21 expression is regulated, particularly in the context of autoimmune diseases, are not yet fully understood.

## 3. Targets of miR-21 Associated with Immunity and Autoimmunity

The majority of miR-21 target genes are tumor suppressors, which are involved in cell proliferation, activation, and apoptosis. Among them, some play important roles in the immune response and the pathogenesis of autoimmune diseases (Table 2).

miR-21 plays important roles in T cell biology. In addition to inhibiting the apoptosis of T cells through the downregulation of PDCD4 [4,14], TIPE2 [7], and FASL [15], miR-21 promotes the activation of T cells through the downregulation of its target SPRY1 [5]. However, miR-21 is also able to inhibit T cell activation through the downregulation of GNAQ, PLEKHA1, and CXCR4 [16]. The discrepancy of these results could be caused by the use of CD4^+^ lymphocytes from different species (human or mouse) and the strategies to modulate miR-21 expression (miR-21 mimic or lentiviral transduction). In addition to regulate T cell apoptosis and activation, miR-21 could also regulate T helper cell differentiation. It has been shown that miR-21 can promote Th2 cell differentiation through the downregulation of its target gene IL-12p35 in dendritic cell [17] and Th17 cell differentiation through the downregulation of SMAD7 in T cells [18], which is a negative regulator of Th17 differentiation. CD70 and LFA-1 are closely linked to the functions of memory T cells. miR-21 promotes the expression of those two molecules via targeting RAS guanyl-releasing protein 1 (RASGRP1) in T cells [19]. miR-21 can also downregulate the expression of CCR7 [20], a molecule involved in homing of T cells to various secondary lymphoid organs.

In addition to regulating the T cell response, miR-21 also plays important roles in myeloid cell function. miR-21 can promote the differentiation of dendritic cells (through the down-regulation of JAG1 [21]) and inhibit dendritic cell maturation [22]. Our published results showed that miR-21 negatively regulates the TLR4 signaling pathway through the downregulation of its target PDCD4, which leads to the promotion of IL-10 expression and blocking of NF-κB activity [4]. miR-21 enhanced the frequency of cytokine-induced myeloid-derived suppressor cell (MDSC) via targeting PTEN [23], a phosphatase that can directly inhibit the activity of PI3K. TIMP3 is a metalloproteinase inhibitor that inhibits the degradation of extracellular matrix. A recent study showed that miR-21 in epidermal keratinocytes promotes the expression of inflammatory factors and the development of autoimmune psoriasis through the downregulation of TIMP3 [24].

## 4. miR-21 as a Diagnostic Biomarker for Autoimmune Disease

miRNAs are important biomarkers in the diagnosis and prognosis of cancer. Recent studies indicate that they can also be used as biomarkers for autoimmune disease. However, whether miR-21 can be used as a diagnostic biomarker for autoimmune disease is still contradictory (Table 3). It has been shown that expression of miR-21 was significantly increased in the plasma, peripheral blood mononuclear cells (PBMC), B cells, and CD4^+^ T cells in patients with SLE [25,26,27]. However, both up and downregulation of miR-21 were detected in the plasma, PBMC, and synovial fluid CD4^+^ T cells in RA patients [26,28,29]. It remains to be determined whether this discrepancy was caused by sample source, detection methods, or stages of the development of the disease. miR-21 expression was significantly upregulated in PBMC and brain lesions of patients with multiple sclerosis [30], as well as in human psoriatic skin lesions [18,31]. In addition, The expression of miR-21 was significantly upregulated in PBMC of patients with T1D [32]. Therefore, miR-21 may serve as a diagnostic and prognostic marker for MS, psoriasis and T1D. Crohn’s disease (CD) and ulcerative colitis (UC) are two major clinical forms of inflammatory bowel disease (IBD). One study found that miR-21 was over-expressed in the serum and mucosa of patients with UC [33]. However, Schaefer *et al.* found that the expression of miR-21 significantly decreased in the blood of patients with UC and CD [34]. Interestingly, upregulation of miR-21 was detected in colon and saliva from UC, but not CD patients [34]. This indicates that miR-21 may be used for the differential diagnosis of UC and CD patients. In addition, miR-21 expression was significantly upregulated in the bronchial epidermal cells of patients with asthma [35] and type-1 autoimmune hepatitis [36].

In summary, although miR-21 is significantly upregulated or downregulated in several autoimmune diseases, it remains to be determined whether it can be used as a biomarker for the diagnosis and prognosis of autoimmune disease.

## 5. Mechanisms by Which miR-21 Regulate the Development of Autoimmune Diseases

Emerging studies have uncovered dysregulated miR-21 expression during the natural course of autoimmune diseases, suggesting that changes in miR-21 levels may contribute to the molecular mechanisms of these diseases. miR-21 can modulate T cell activation and apoptosis, Th17 cell differentiation, Treg cell development and function, Th1/Th2 balance, DNA hypomethylation, and pancreatic β cell apoptosis.

### 5.1. miR-21 and T Cell Activation

Activation of self-reactive T cells contributes to the development of autoimmune diseases. miR-21 expression was significantly increased in activated Jurkat cells and primary CD4^+^ T lymphocytes compared with that in non-stimulated cells [29]. Further study showed that miR-21 promotes ERK and JNK signaling in activated T cells through the downregulation of Sprouty1. Consistent with these results, miR-21 over-expression promotes activator protein 1 (AP-1) activity and interleukin-2 (IL-2) expression. These results suggest that miR-21 may augment the T cell response [5]. miR-21 was upregulated and strongly correlated with SLE disease activity [25]. Silencing of miR-21 reversed the activated phenotype of T cells from patients with SLE. Over-expression of miR-21 in normal T cells led to acquisition of an activated phenotype through the downregulation of PDCD4 [25]. However, another study showed that miR-21 over-expression in Jurkat cells results in lower ERK phosphorylation, AP-1 activation and CD69 expression [16]. miR-21 inhibition in primary human lymphocytes promotes IFN-γ production and activation in response to T-cell receptor (TCR) engagement. Further study showed that miR-21 negatively regulates T cell activation through the downregulation of GNAQ, PLEKHA1, and CXCR4 [16]. It is speculated that miR-21 expression could play a role in modulating TCR sensitivity of memory T-cells to avoid hyper-activation in response to sub-optimal stimulation. Further studies will be necessary to validate this hypothesis.

### 5.2. miR-21 and T Cell Apoptosis

miR-21 inhibits the apoptosis of T cells by targeting tumor suppressors. Recently we found that Tipe2, a well-known tumor suppressor, provides a molecular bridge between miR-21 and cell apoptosis [7]. miR-21 suppresses the apoptosis of activated T cells through directly targeting tumor suppressor TIPE2. TIPE2 expression was downregulated in peripheral blood mononuclear cells from patients with systemic lupus erythematosus [38], which indicates that the miR-21-TIPE2 axis may play important roles during the pathogenesis of SLE. Treg cells displaying a memory phenotype were abundant in the synovial fluid (SF) of RA patients. These Treg cells demonstrate little apoptosis which could be due to increased expression of miR-21 in SF Treg cells [39]. miR-21 was significantly upregulated in psoriasis skin lesions. Specific inhibition of miR-21 increased the apoptosis rate of activated T cells and over-expression of miR-21 may contribute to T cell-derived psoriatic skin inflammation [31].

### 5.3. miR-21 and Th17 Cell Differentiation

Th17 cells secrete IL-17, IL-21, IL-22, and other pro-inflammatory cytokines and play an important role in the pathogenesis of many autoimmune diseases, such as MS or experimental allergic encephalomyelitis (EAE), psoriasis, and T1D. In T cells, miR-21 promotes the proliferation of T cells and the production of IFN-γ and IL-17 through the down-regulation of PDCD4 [18]. Recently, Murugaiyan *et al.* found that miR-21 expression is elevated in Th17 cells and that miR-21-deficient mice produce fewer Th17 cells and are resistant to EAE [18]. Further studies showed that miR-21 promotes Th17 differentiation by targeting SMAD-7, a negative regulator of TGF-β signaling. miR-21 promotes the activation of SMAD-2/3, suppresses IL-2 expression, and ultimately enhances the activity of the TGF-β signaling pathway and promotes the differentiation of Th17 cells. However, Dong *et al.* have found that in the PBMC of RA patients, the expression of miR-21 was decreased while accompanied with increased expression of inflammatory factors such as IL-17, IL-22, and TNF-α and the transcription factor STAT3 [28]. Therefore, the authors speculate that miR-21 may negatively regulate Th17 cell differentiation. Trough integrative computational mRNA–miRNA interaction analyses of the autoimmune-associated miRNAs and well-known factors that regulate Th17 differentiation, Honardoost *et al.* attempted to discover new potential miRNAs that are involved in Th17 differentiation [40]. Consistent with the study by Dong *et al.*, they found that miR-21 may inhibit Th17 cell differentiation by targeting the Th17 positive regulatory factor STAT3. SMAD-7 is a direct target of miR-21 and negatively regulates the activity of TGF-β signaling. However, whether SMAD-7 promotes or inhibits Th17 cell differentiation is still elusive. TGF-β signaling is essential for both Th17 and Treg cell differentiation. SMAD-7 may inhibit Th17 cell differentiation through the suppression of TGF-β signaling pathway. However, SMAD-7 may also promote Th17 differentiation through the suppression of Treg cell differentiation since there is a reciprocal relationship between pro-inflammatory Th17 and anti-inflammatory Treg cells. Nevertheless, the exact molecular mechanism by which miR-21 regulates Th17 cell differentiation remains to be elucidated.

### 5.4. miR-21 and Treg Development/Function, Th1/Th2 Balance, and DNA Hypomethylation

Regulatory T cells (Tregs) are essential to the maintenance of self-tolerance. Defective Tregs has been shown to contribute to the break of tolerance in human autoimmune disease. miR-21 expression was upregulated in human natural Tregs. Furthermore, miR-21 acted as a positive, though indirect, regulator of FOXP3 expression [41]. The frequency of Treg cells and the level of miR-21 were significantly lower in PBMC from RA patients. This was accompanied by the increase in STAT3 expression and activation, and a decrease in STAT5/pSTAT5 protein and Foxp3 mRNA levels [28]. However, another study showed that, although miR-21 level was upregulated in Tregs, inhibition of miR-21 in Tregs did not alter FoxP3 expression [42]. Because of this, miR-21 expression was postulated to be a marker of memory cells but not a Treg-specific marker. Ectopic expression of miR-21 led to increased Gata3 expression and decreased expression of Sprouty1, thus promoted Th2 differentiation [8]. Increased miR-21 expression in CD4^+^ T cells from both lupus patients and lupus-prone MRL/lpr mice promoted cell hypomethylation by repressing RAS guanyl-releasing protein 1 (RASGRP1) expression. This, in turn, leads to the downregulation of DNA methyltransferase 1 (DNMT1) expression and over-expression of autoimmune-associated methylation-sensitive genes, such as CD70 and LFA-1, via demethylation of their promoters [19].

### 5.5. miR-21 Regulates Myeloid Cell Function

In addition to regulate T cell function, miR-21 also plays an important role in the differentiation and the immune response of dendritic cells and macrophages. Blocking the expression of miR-21 can prevent the differentiation of human monocyte-derived dendritic cells [43]. In addition, miR-21 plays a central role in establishing the balance of Th1 *versus* Th2 responses to antigens through targeting IL-12p35 in dendritic cell [17]. Uncontrolled inflammation is often associated with autoimmune diseases. Our research work revealed an important role of miR-21 during this process, *i.e.*, miR-21 negatively regulates TLR4 signaling through the targeting of PDCD4 in macrophages [4].

### 5.6. miR-21 and β Cell Apoptosis

miR-21 could be involved in the pathogenesis of autoimmune diseases by not only regulating the function of immune cells but also the inhibition of apoptosis of non-immune cells. Apoptosis of pancreatic β cells is a critical component of the pathological process of T1D. It has been reported that antagonizing miR-21 expression can promote the apoptosis of mouse pancreatic islet cell line MIN6 [44]. However, it has also been shown that, although increased expression of miR-21 can promote the proliferation of inflammatory factor-treated mouse β cell line INS-1, it also promotes the synthesis of nitric oxide (NO) and the apoptosis of those cells [45]. A study from our group showed that NF-κB induces the expression of miR-21 in mouse β cell line β-TC-6. Increased miR-21 expression leads to the downregulation of PDCD4 and protects cells from Bax family-mediated apoptosis [14]. Deficiency of PDCD4 was found to protect β cells from streptozotocin-induced cell death, and conferred resistance to spontaneous autoimmune diabetes in non-obese diabetic (NOD) mice. Thus, NF-κB-miR-21-PDCD4 axis plays a crucial role in the development of T1D and represents a unique therapeutic target for treating the disease. However, the exact role of miR-21 in diabetic research remains elusive. Further studies related to validation and elucidation of the mechanism by which miR-21 and its targets impact are necessary.

## 6. miR-21 as a Therapeutic Target for the Treatment of Autoimmune Diseases

In recent years, miRNA has become the attractive target for disease therapy. Currently miRNA drugs have entered clinical trials for the treatment of virus infection, cancer, heart disease, and diabetes [46]. Since miR-21 regulates the activation, differentiation, and apoptosis of immune cells, and plays an important role in the development of autoimmune disease, it may become an important therapeutic target for the treatment of autoimmune disease.

Murugaiyan *et al.* reported that inhibition of miR-21 expression using a tiny seed-targeting locked nucleic acid (LNA)-antimiR led to the downregulation of Th17 cell-related cytokines, such as IL-17A, IL-17F, IL-21, and IL-22, and can effectively alleviate the development and progression of EAE in mice [18]. Guinea-Viniegra *et al.* also showed that antagonizing miR-21 reduced psoriasis pathology in both a psoriasis-like mouse model and mouse xenotransplantation model [24]. Upon miR-21 inhibition, mRNA expression of miR-21 targets, such as TIMP-3 and PDCD4, were upregulated, whereas TNF-α, IL-17, and IL-21 mRNA was downregulated. Garchow *et al.* have shown that silencing of microRNA-21 *in vivo* ameliorates autoimmune splenomegaly in a spontaneous genetic mouse model of SLE [47]. Further study showed that treatment with anti-miR-21 altered CD4/CD8 T cell ratios and reduced Fas receptor-expressing lymphocytes. In 2016, the same group reported that miR-21 deficiency reduced lupus-like autoimmunity in the murine chronic graft-*versus*-host disease model (cGVHD) of SLE [48]. cGVHD host mice with miR-21deficiency exhibited significantly reduced splenomegaly and autoantibody titers. Components of the CD40:CD40L and CD28:CD80/86 co-stimulation pathways were also found to be downregulated. In addition, miR-21-deficient hosts showed reduced number of CD4^+^ IL-17^+^ cells but increased number of CD4^+^ CD25^+^ Foxp3^+^ cells.

The results showed that, although the precise molecular mechanism of miR-21 mediated autoimmune disease is unknown and most of the work was done using mouse models of human autoimmune disease, miR-21 is a promising therapeutic target for the treatment of autoimmune disease. However, it must be noted that miR-21 in different organs and cell types may play different or opposite roles during the development of autoimmune disease. Systemic drug administration may result in poor therapeutic effect and side effects. Therefore, it is essential to determine the role of miR-21 in specific tissues or cell types before it can be used as a therapeutic target for the treatment of autoimmune disease.

## 7. Concluding Remarks

There is no doubt that miR-21 plays an important role in the development of autoimmune disease. However, there are still many contradicting reports on the role of miR-21 during the pathogenesis of autoimmune disease. Further studies are necessary to determine the target genes in different cell types and the molecular mechanisms underlying the pathogenesis of different autoimmune diseases before miR-21 can be used in therapeutic applications.

## Figures and Tables

**Table 1:** List of well-known positive and negative regulators of miR-21 expression.

Positive Regulators	References	Negative Regulators	References
AP-1	[11]	Bcl-6	[8]
STAT-3	[12]	GAS5	[9]
NF-κB	[7,13]	PAPD5	[10]

**Table 2:** Targets of miR-21 associated with immunity and autoimmunity.

Name	Description	Cell Types	Function	Refs.
PDCD4	Programmed cell death 4	T cell	Inhibits T cell apoptosis	[14]
			Promotes T cell activation	[25]
		Macrophage	Promotes IL-10 production	[4]
SPRY1	Sprouty homolog 1	T cell	Promotes T cell activation	[5]
IL-12p35	Interleukin 12, subunit p35	DC	Inhibits Th1 differentiation	[17]
SMAD-7	Sma and Mad (Mothers against decapentaplegic) 7	T cell	Promotes Th17 differentiation	[18]
TIPE2	TNF-α-induced protein 8 (TNFAIP8)-like 2	T cell	Inhibits T cell apoptosis	[7]
PTEN	Phosphatase and tensin homolog	MDSC	Promotes MDSC expansion	[23]
RASGRP1	RAS guanyl-releasing protein 1	T cell	Promotes hypomethylation	[19]
CCR7	Chemokine C receptor 7	DC	Inhibits DC maturation	[22]
		T cell	Reduces T cell homing	[20]
JAG1	Jagged 1	DC	Promotes DC differentiation	[21]
TIMP3	Tissue inhibitor of metalloproteinase 3	Epidermal keratinocytes	Promotes inflammation	[24]
PLEKHA1	Pleckstrin homology domain-containing family A member 1	T cell	Inhibits T cell activation	[16]
GNAQ	Guanine nucleotide-binding protein G(q) subunit alpha	T cell	Inhibits T cell activation	[16]
CXCR4	C-X-C chemokine receptor type 4	T cell	Inhibits T cell activation	[16]
FASL	Fas ligand	MCF-7	Inhibits T cell apoptosis	[15]

Abbreviations: MDSC, Myeloid-Derived Suppressor Cell; DC, Dendritic Cell; MCF-7, Breast cancer cell line.

**Table 3:** miR-21 as a diagnostic biomarker for patients with autoimmune disease.

Disease	Cells or Tissue	Expression	Refs.
SLE	plasma, PBMC, CD4^+^ cells	up-regulation	[25,26,27]
RA	plasma, memory CD4^+^ cells	up-regulation	[26,29]
RA	PBMC, CD4^+^ cells	down-regulation	[28]
MS	PBMC, brain lesions, CD4^+^ cells	up-regulation	[30,37]
Psoriasis	skin lesions	up-regulation	[18,31]
T1D	PBMC	up-regulation	[32]
UC	serum, mucosa, colon, saliva	up-regulation	[33,34]
UC, CD	blood	down-regulation	[34]
Asthma	bronchial epidermal cells	up-regulation	[35]
AIH-1	serum	up-regulation	[36]

Abbreviations: SLE, Systemic Lupus Erythematosus; RA, Rheumatoid Arthritis; MS, Multiple Sclerosis; T1D, type 1 diabetes; UC, ulcerative colitis; CD, Crohn’s disease; AIH-1, type-1 autoimmune hepatitis; PBMC, peripheral mononuclear cells.
